# Multiple genetically engineered humanized microenvironments in a single mouse

**DOI:** 10.1186/s40824-016-0066-2

**Published:** 2016-06-28

**Authors:** Jungwoo Lee, Dirk Heckl, Biju Parekkadan

**Affiliations:** Department of Surgery, Center for Engineering in Medicine, Massachusetts General Hospital & Harvard Medical School and Shriners Hospital for Children, Boston, MA USA; Department of Medicine, Brigham and Women’s Hospital, Boston, MA USA; Harvard Stem Cell Institute, Cambridge, MA USA; Department of Chemical Engineering, Institute for Applied Life Sciences, University of Massachusetts, Amherst, MA USA

**Keywords:** Genetically engineered stroma, Chimeric mouse model, Human hematopoietic stem and progenitor cells, Implantable microenvironments, Scaffolds, Bone marrow stromal cells

## Abstract

**Background:**

Immunodeficient mouse models that accept human cell and tissue grafts can contribute greater knowledge to human stem cell research. In this technical report, we used biomaterial implants seeded with genetically engineered stromal cells to create several unique microenvironments in a single mouse. The scope of study was focused on human CD34 hematopoietic stem/progenitor cell (HSPC) engraftment and differentiation within the engineered microenvironment.

**Results:**

A mouse model system was created using subdermal implant sites that overexpressed a specific human cytokines (Vascular Endothelial Growth Factor A (hVEGFa), Stromal Derived Factor 1 Alpha (hSDF1a), or Tumor Necrosis Factor Alpha (hTNFa)) by stromal cells in a three-dimensional biomaterial matrix. The systemic exposure of locally overexpressed cytokines was minimized by controlling the growth of stromal cells, which led to autonomous local, concentrated sites in a single mouse for study. This biomaterial implant approach allowed for the local analysis of each cytokine on hematopoietic stem cell recruitment, engraftment and differentiation in four different tissue microenvironments in the same host. The engineered factors were validated to have bioactive effects on human CD34+ hematopoietic progenitor cell differentiation.

**Conclusions:**

This model system can serve as a new platform for the study of multiple human proteins and their local effects on hematopoietic cell biology for in vivo validation studies.

**Electronic supplementary material:**

The online version of this article (doi:10.1186/s40824-016-0066-2) contains supplementary material, which is available to authorized users.

## Background

Immunodeficient mice lack immune cell activity and do not acutely reject xenogenic cells and tissues [1, 2]. These mouse strains have been an enabling tool for understanding human cell growth and functions in the context of living systems [3–6]. In particular, these mouse models have greatly advanced functional characterization of human hematopoietic stem and progenitor cells (HSPCs) [7, 8]. Human HSPCs can be intravenously injected and engrafted into a host immunodeficient mouse to form a chimeric hematopoietic system. These experiments have helped to identify and validate factors that control human HSPCs migration, engraftment, self-renewal, and differentiation in mice for mechanistic and therapeutic purposes [9]. Yet, there still remain critical areas to improve these immunodeficient mouse models to better model human HSPCs [10]. For example, human HSPCs do not engraft into a human stromal cell bed which may be important for long-term support of human stem cell function by direct physical contact or the local secretion of stromal soluble factors.

Several approaches have been tested in order to improve the functional support of human HSPCs in immunodeficient mice. First, human cytokines have been directly injected to a host mouse before and after human HSPCs injection that promoted engraftment and differentiation of human cells when compared to the control [11, 12]. Since single injection of human cytokines only produces a transient effect, repeated injections are typically required, which is cost-prohibitive and burdensome. Alternatively, human gene encoded lentiviral vectors [13] or plasmid deoxyribonucleic acids (DNAs) [14] have been applied to directly transfect host cells and synthesize human cytokines. Second, using gene knock-in methods, immunodeficient mouse strains have been generated to express human major histocompatibility complex (MHC) while suppressing mouse MHC molecules that improved human immune system development [2, 15]. Transgenic expression of human cytokines and growth factors has been also demonstrated to modulate human HSPCs responses [14]. For instance, human stem cell factor, granulocyte-macrophage colony stimulating factor and interleukin-3 genes inserted NOD-scid IL2rγ^null^ (NSG) mice models demonstrated enhanced hematopoietic reconstitution of human HSPCs [16]. Although this approach is promising, it is labor/time-intensive, requires specialized facilities and equipment, and is limited in throughput of genes expressed in a single mouse. Moreover, ubiquitous expression of human molecules could be problematic to interpret experimental outcomes. Lastly, human cells and tissues can be directly introduced into the murine host. For example, human bone marrow stromal cells (hBMSCs) were introduced into a mouse bone marrow cavity by intra-femoral injection. The presence of human stromal cells in a mouse bone marrow increased engraftment of subsequently injected human HSPCs [17]. However, this invasive procedure is compatible with only a few bones which limits the throughput of this approach in supporting human HSPCs function. Human fetal thymic and fetal liver tissues, known sites of early hematopoiesis, have been implanted in an immunodeficient mouse prior to human HSPCs injection. A mouse carrying primary human fetal tissue organoids demonstrated complete hematopoietic reconstitution of human HSPCs [18]. Although this study demonstrated the possibility to recreate a full spectrum human hematopoietic cells in mouse models, human fetal thymus and liver tissues are difficult to widely source making broad adoption unattainable.

In this report, we introduce an integrated bioengineering approach that can increase the throughput of model systems research for human HSPCs by using genetically engineered scaffold microenvironments. Engineered stromal cells, stably synthesizing a human cytokine or growth factor, were embedded in three-dimensional (3D) porous hydrogel scaffolds and implanted into immunodeficient mice in different subcutaneous locations. Each implant created a unique hematopoietic-supportive tissue microenvironment over time. These implants exhibited a locally concentrated environment of human cells and soluble factors with the opportunity to make multiple, autonomous scaffolds implanted into the same mouse. We demonstrated the engineering of stromal cells with individual human cytokines, the control of local and systemic exposure of engineered cytokines, and functional characterization of the implanted microenvironments with human HSPCs. The presented method is versatile and can be readily applied to other human cells and cytokines with minor modifications for better understanding human biology.

## Methods

All chemicals and supplies were purchased from Sigma Aldrich or Fisher Scientific unless otherwise stated. All mouse and primary human cell experiments were reviewed and approved by an internal review board of Massachusetts General Hospital.

### Stromal cell culture

Primary hBMSCs were isolated from healthy donor’s fresh bone marrow (Lonza) following a previously reported protocol [19]. Briefly, hBMSCs were cultured with medium composed of 15 % fetal bovine serum, 100 U/mL penicillin, 100 μg/mL streptomycin, 20 mg/L gentamicin, 1 ng/L fibroblast growth factor, and 3 g/L sodium bicarbonate in alpha-minimum essential medium (a-MEM). Human umbilical vein endothelial cells (HUVECs) were purchased from Invitrogen and cultured with medium composed of 10 % low serum growth supplement, 100 U/mL penicillin and 100 μg/mL streptomycin. Mouse bone marrow stromal cells (mBMSCs) were purchased from Invitrogen and cultured with medium composed of 15 % fetal bovine serum, 100 U/mL penicillin, 100 μg/mL streptomycin, 20 mg/L gentamicin, 1 ng/L fibroblast growth factor, and 3 g/L sodium bicarbonate in a-MEM. All cell cultures were maintained at 37 °C, 5 % CO_2_ and 100 % humidity. Human peripheral blood derived CD34 cells were purchased from StemCell Technologies and after thawing immediately used for in vivo experiments without culture.

### Generation of lentiviral particles encoded human VEGFa, SDF1a and TNFa genes

The self-inactivating lentiviral vector backbone pRRL.PPT.SFFV.IRES.eGFP were kindly provided by Christopher Baum (Hannover Medical School, Hannover, Germany). cDNA clones of the human cytokines Vascular Endothelial Growth Factor A (hVEGFa), Stromal Derived Factor 1 Alpha (hSDF1a), and Tumor Necrosis Factor Alpha (hTNFa) were purchased from Open Biosystems, Polymerize chain reaction (PCR) amplified, and cloned into pRRL.PPT.SFFV.IRES.eGFP. Lentiviral particles were generated by transient calcium phosphate transfection of HEK293T cells (ATCC CRL-3216) with the lentiviral constructs, psPAX2 (Addgene Plasmid #12260), and pMD2.g (VSV-G, Addgene Plasmid #12259) at the ratio 1: 2: 0.33. Viral supernatants were harvested 40 h post transfection by centrifugation and stored −80 °C until use.

### Generation of genetically engineered mouse bone marrow stromal cells

mBMSCs were plated in a 12-well plate. Once the culture reached about 50 % confluence, the medium was replaced with 0.5 ml of new medium containing 1:10 diluted lentiviral particles and 2 μg/ml Polybrene (Hexadimethrine bromide), and incubated for 8 h. Afterwards the cell surface was washed with PBS three times and 1 ml of normal medium was added in each well. After 48 h, mBMSCs expressing green fluorescent protein (GFP) were sorted out using a fluorescence activated cell sorting (FACS) machine (BD FACSAria II cell sorter). Expression of GFP was confirmed under a fluorescent microscope (Zeiss Axio 200) and secretion of hVEGFa, hSDF1a, and hTNFa was determined by enzyme-linked immunosorbent assay (ELISA) kits (R&D systems).

### Fabrication of inverted colloidal crystal hydrogel scaffolds and in vitro 3D stromal cell culture

Polyacrylamide hydrogel based inverted colloidal crystal scaffolds were prepared following the previously reported methods [20]. Final hydrogel scaffolds were consisted of regularly arranged spherical pores (D = 250 ± 30 μm) of which surface was coated with type I collagen utilizing amine reactive heterbifunctional cross-linker (Sulfo-SANPH). Dimensions of cylindrical shape hydrogel scaffolds were about 1.5 mm in thickness and 6.5 mm in diameter. Prior to cell seeding, hydrogel scaffolds were dehydrated in a laminar hood for 30 min and then a dense genetically engineered mBMSCs or hBMSCs (0.5 × 10^6^ cells in 40 μl) was dropped on the hydrogel scaffold. Rehydration process promoted effective cell distribution into a complex 3D porous geometry and seeded stromal cells formed stable adhesion on collagen fiber coated pore surface in 4–6 h. After 3 days culture, the level of hVEGFa, hSDF1a, and hTNFa molecules in the medium was determined using ELISA kits. Total cell mass in each scaffold was quantified using a MTT 3-(4,5-dimethylthiazol-2-yl)-2,5-diphenyltetrazolium bromide (MTT) reagent (ATCC) and used for normalization of cytokine secretion.

### In vivo microenvironments formation with genetically engineered stromal cells

Three different stromal compositions were used for creating subcutaneous microenvironments in single host mouse (Table 1); (i) Growth-competent genetically engineered mBMSCs (0.5 × 10^6^ cells in 40 μl); (ii) 1:1 mixture of growth-competent genetically engineered mBMSCs (0.25 × 10^6^ in 20 μl) and hBMSCs (0.25 × 10^6^ in 20 μl); (iii) Growth-arrested genetically engineered mBMSCs (0.5 × 10^6^ in 40 μl). Growth-arrested engineered stromal cells were prepared by treating growth competent stromal cells with 10 μg/mL mitomycine C containing medium for 3 h. Post 3-day in vitro culture, stromal cell-scaffolds were subdermally implanted on the dorsal side of a 8 week-old NOD/SCID/IL2ν^null^ (NSG) male mouse (Jackson Laboratory) following the previously reported method [20]. Four types of cell-scaffolds were implanted beneath the skin of immunodeficient mice. A distance between implanted scaffolds was maintained at least 2 cm in order to prevent their contact. After 6 weeks, mice were scarified and peripheral blood (0.5–1 ml) and implanted scaffolds samples were retrieved. Bloods were allowed to clot at room temperature for 1 h, centrifuged at 6000 rpm for 10 min, and serum was collected. The level of hVEGFa, hSDF1a, and hTNFa molecules in serum was determined using ELISA kits. A low level of hSDF1a was detected in control NSG mice without carrying scaffolds due to antibody cross-reactivity with mouse SDF1a. This background signal was subtracted from the final data. Explanted scaffolds were embedded in optical cutting temperature compound, snap frozen with dry ice-chilled 2-methylbutane, and saved for histological analysis.

**Table 1:** Three stromal cell-seeding compositions for creating 4 distinct microenvironments in a mouse

	Composition A	Composition B	Composition C
Implant site 1	Growth-competent mBMSCs secreting hVEGFa	Growth-competent mBMSCs secreting hVEGFa + hBMSCs	Growth-arrested mBMSCs secreting hVEGFa
Implant site 2	Growth-competent mBMSCs secreting hSDF1a	Growth-competent mBMSCs secreting hSDF1a + hBMSCs	Growth-arrested mBMSCs secreting hSDF1a
Implant site 3	Growth-competent mBMSC ssecreting hTNFa	Growth-competent mBMSCs secreting hTNFa + hBMSCs	Growth-arrested mBMSCs secreting hTNFa
Implant site 4	Growth-competent mBMSCs control	Growth-competent mBMSCs control + hBMSCs	Growth-arrested mBMSC control

### Systemic migration of human CD34 cells to the implanted microenvironments

A 2:1 ratio mixture of growth-arrested genetically engineered mBMSCs (0.5 × 10^6^ in 40 μl) and hBMSCs (0.25 × 10^6^ in 20 μl) was introduced into a scaffold. Post 3 days in vitro culture, the cell-scaffolds were subcutaneously implanted into NSG mouse. After 4 weeks implantation, 2.5 × 10^5^ human CD34 (hCD34) cells were intravenously injected. Post 24 and 72 h, mice were sacrificed and implanted scaffolds, bone marrow and spleen samples were collected. Hematopoietic cells were retrieved from scaffold and tissue samples following the previously reported method [20]. Briefly, explanted scaffolds were minced into small pieces (1–2 mm), incubated in 2 mg/ml collagenase solution for 20 min and thoroughly mashed using a plunger rod from a 3 ml syringe. 40 μm cell-strainers were used to separate cells from hydrogel scaffold and tissue debris. For bone marrow cell isolation, tibias and femurs were dissected and the marrow space was flushed with medium using a 23-gauge needle. Collected marrow cells were passed through 70 μm cell-strainers to remove bony spicules and debris and treated with red blood cell (RBC) lysis buffer for 1–2 min. For spleen cell isolation, a retrieved spleen was placed on a 40 μm cell-strainer and gently mashed using a plunger rod with Rosewell Park Memorial Institute (RPMI) medium. Harvested spleen cells were treated with RBC lysis buffer for 2–3 min. Retrieved hematopoietic cells were stained with fluorescein isothiocyanate (FITC) conjugated anti-human CD34 antibody (BD Pharmingen) and analyzed using FACS machine (BD) and FlowJo program.

### Direct implantation of human CD34 cells with genetically engineered stromal cell-scaffolds and ex vivo methylcellulose assay

A 2:1 ratio mixture of growth-arrested genetically engineered mBMSCs (0.5 × 10^6^ in 40 μl) and hBMSCs (0.25 × 10^6^ in 20 μl) was introduced into a scaffold. Post 3 days culture, cell-scaffold plates were placed in a refrigerator for 20 min and then removed medium both from a well and a hydrogel scaffold using a pipette. Next ice-cold, moderately dehydrated cell-scaffolds were transferred to a 96-well plate and 50 μl of ice-cold matrigel containing the mixture of 2 × 10^5^ HUVECs and 2 × 10^5^ hCD34 cells was infiltrated into the hydrogel scaffold pores via centrifugation at 4 °C, 1500 rpm for 15 min. The matrigel-cell filled scaffolds were incubated for 20 min at 37 °C incubator to solidify the gel and then 1 ml of culture medium was placed in each well.

After 3 weeks in vivo implantation, hematopoietic cells were retrieved from explanted scaffolds following the above procedure. Hematopoietic cells were dispersed in methylcellulose medium containing recombinant cytokines and erythropoieting for human cells (MethoCult H4034, Stem Cell Technologies). Final concentration was 1 × 10^5^ hematopoietic cells in 1.1 ml methylcellulose medium that was plated in a 35 mm dish following a protocol provided by the vendor. After 2 weeks incubation, colony forming units of (i) erythroid, (ii) granulocyte and macrophage, (iii) granulocyte, erythrocyte, monocyte and megakaryocyte, and (iv) burst forming unit of erythroid were distinguished and counted under an inverted light microscope.

### Histological analysis

Frozen scaffold blocks were cut into 10–30 μm thickness using a cryostat (Leica Biosystems) and stored at −80 °C until use. For hematoxylin and eosin, and Masson’s Trichrome staining, frozen tissue sections were fixed with 10 % buffered formalin solution and stained following the vendor’s protocol (American Master Tech). For immunofluorescence staining, frozen tissue sections were fixed with ice-cold acetone, blocked with 10 % normal goat serum and 1 % bovine serum albumin diluted in phosphate buffered saline (PBS). Slides were incubated with rat anti-mouse CD31 (BD Pharmingen) and rabbit anti-human CD34 antibody (Abcam) for overnight. Subsequently the slides were stained by goat anti-rat immunoglobulin G (IgG) conjugated with alexa fluor 488 and goat anti-rabbit IgG conjugated with alexa fluor 568 (Invitrogen) for 1 h. Finally, VectaShield mounting medium with DAPI was applied and slides were imaged under a fluorescence (Zeiss 200) microscope. Open source image analysis software, ImageJ, was used to process and quantify areas of collagen fibers and vasculatures in Masson’s Trichrome and mouse CD31 stained slides, respectively.

### Imaging

Explanted scaffolds were fixed in 2 % glutaladehyde solution for 6 h and then serially dehydrated with 20, 50, 70, 90 and 100 % ethanol solution. The scaffolds were further dried using a lyophilzer overnight. A thin platinum/gold coating was made on the samples using a sputter coating machine (208HR,Cressington) and imaged under FESEM Ultra55 (Zeiss).

### Statistics

Statistical comparisons of data were per- formed using SPSS version 17 software. Nonparametric tests, i.e., Kruskal-Wallis and Mann-Whitney tests, were applied for ELISA and LSK cell analysis, respectively. Comparisons of human cell engraftment in long-term engraftment studies were performed using an unpaired Student *t* test on GraphPad PRISM version 5.

## Results

### Genetically engineered mouse stromal cell lines secreting human VEGFa, SDF1a, or TNFa

**Fig. 1:**
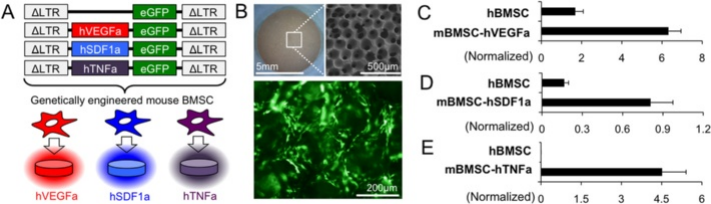
Creating genetically engineered stromal cell-coated implantable microenvironments. **a** Design of lentiviral vectors encoding hVEGFa, hSDF1a, and hTNFa genes for genetically engineered mBMSC-line generation. b Microfabricated hydrogel scaffold that represents a standardized and fully interconnected porous microstructure (*top*) and a fluorescent image of genetically engineered mBMSC residing in a 3D scaffold (*bottom*). **c**-**d** Normalized secretion of (**c**) hVEGFa, (**d**) hSDF1a, and (**e**) hTNFa from genetically engineered stromal cells for 3 days. The secretion rates were compared with hBMSC growing in the same hydrogel scaffolds

In order to create a specific human soluble factor enriched microenvironment, we first designed lentiviral vectors that encoded human vascular endothelial growth factor a (hVEGFa), human stromal cell derived factor-1 alpha (hSDF1a), and human tumor necrosis factor alpha (hTNFa) genes along with enhanced green fluorescent protein (eGFP) (Fig. 1a). A lentiviral control was also applied expressing eGFP but not a specific cytokine. mBMSCs were infected with lentiviral particles and sorted by FACS to purify eGFP cells. Mouse cells were used for these studies to ensure long-term survival of engineered stromal cells because even severely immuncompromised mice still retain immune compartments that can detect human cells. The purified cells were culture-expanded to establish 3 genetically engineered mBMSC-lines i.e. mBMSC-hVEGFa, mBMSC-hSDF1a, and mBMSC-hTNFa (Additional file 1: Figure S1).

Genetically engineered stromal cells were then seeded into the 3D hydrogel scaffolds following the previously reported methods [20]. These hydrogel scaffolds consisted of regularly arranged spherical cavities, whereby the cavity surfaces were coated with type I collagen. This coating method promoted homogenous stromal cell seeding and subsequent adhesion (Fig. 1b). The characterized rate of soluble factor secretion of genetically engineered stromal cells in the scaffolds was 4.42 ± 0.24 μg/mL for hVEGFa, 0.87 ± 0.16 μg/mL for hSDF1a, and 2.7 ± 0.02 μg/mL for hTNFa over 3 days. When compared to primary hBMSCs growing in the scaffolds, normalized hVEGFa and hSDF1a secretion were about 4.8 and 3.7 folds higher, respectively (Fig. 1c-e). hBMSCs do not naturally secrete hTNFa. These stable cell lines were advanced for further in vivo testing.

### Control of systemic and local exposure of engineered factors after in vivo implantation

**Fig. 2:**
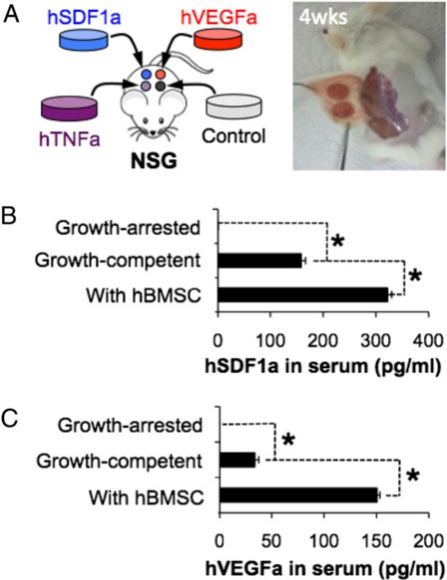
Characterization of human cytokines in peripheral blood of mice carrying different states of genetically engineered stromal cell-laden scaffolds. **a** Schematic of creating different human cytokine producing local microenvironments beneath the skin in an immunodeficient NSG mouse and a gross image of scaffolds after 4 weeks implantation. **b**, **c** Detection of (**b**) hSDF1a and (**c**) hVEGFa in mouse blood serum post 6-week implantation (*N* = 3–5, *P < 0.05). A low level hSDF1a caused by cross-reactivity with mouse SDF1a was subtracted from the data. Systemic level hSDF1a and hVEGFa were detected in genetically engineered growing mBMSC scaffolds bearing mice but not hTNFa. Systemic level availability of hSDF1a and hVEGFa were significantly increased by co-seeding with hBMSC or dramatically decreased by arresting their growth

We subcutaneously implanted genetically engineered growth-competent stromal cell seeded scaffolds into immunodeficient NOD-scid IL2rγ^null^ (NSG) mice and determined whether these engineered factors could be detected in vivo. Four different types of engineered stromal cell-seeded scaffolds were implanted into a NSG mouse (Fig. 2a). Peripheral blood samples were collected at 6 weeks post implantation and the level of human cytokines in serum was measured using ELISA. Detectable levels of hVEGFa (33.93 ± 3.88 pg/ml) and hSDF1a (238.97 ± 8.01 pg/ml) were found in peripheral blood while there was no hTNFa. We next examined whether systemic exposure of secreted molecules can be controlled by manipulating the growth of genetically engineered stromal cells. In our previous studies, hBMSCs accelerated and augmented inter-scaffold angiogenic process via secreting pro-angiogenic and immunomodulatory molecules [20, 21]. To enhance the survival and systemic distribution of secreted molecules, we co-seeded a 1:1 ratio hBMSCs and engineered stromal cells into the scaffolds. Peripheral blood analysis 6 weeks after implantation revealed significantly increased level of hVEGFa and hSDF1a, but again no hTNFa was detected. We then hypothesized that systemic exposure of cytokines secreted from the engineered stromal cells could be reduced by limiting stromal cell proliferation. To test this hypothesis, we treated genetically engineered stromal cells with mitomycine C that bound to microtubules and blocked cellular division. Growth-arrested stromal cells remained viable and maintained comparable levels of human cytokine secretion during 3 weeks of in vitro culture (Additional file 1: Figure S2). Growth arrested stromal cell-seeded scaffolds were subdermally implanted to NSG mice and 6 weeks after peripheral blood levels of human cytokines were measured. We confirmed the absence of hVEGFa and hTNFa, and a lower level of hSDF1a (Fig. 2b, c). The residual hSDF1a level was caused by cross-reactivity of the antibody with mouse SDF1a (Additional file 1: Figure S3). Collectively these results indicate that genetically engineered stromal cells remained bioactive after in vivo implantation. The secretion levels were controllable by manipulating their survival and growth.

### Local tissue formation in scaffolds is altered by stromal cell proliferation

**Fig. 3:**
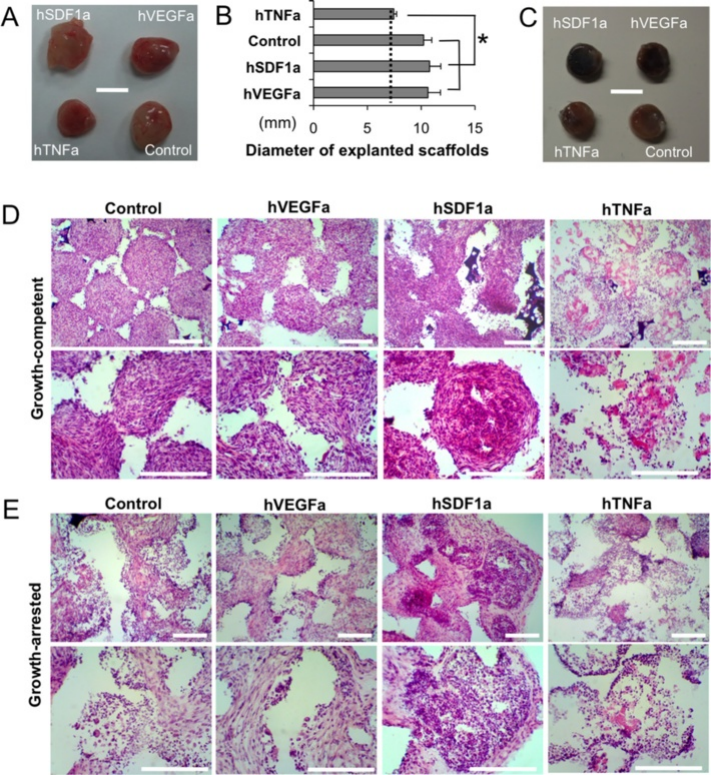
Comparison of tissue formation between growth-competent and growth-arrested genetically engineered stromal cells seeded scaffolds. **a** Gross image of explanted scaffolds with growing genetically engineered stromal cells 6 weeks after implantation, and (**b**) Diameters of explanted scaffolds (*N* = 5, *P < 0.05). **c** Gross image of explanted scaffolds with growth arrested genetically engineered stromal cells 6 weeks after implantation (Scale bar = 5 mm). **d**-**e** Comparison of hematoxylin and eosin stained tissue sections between growth-competent and growth-arrested engineered stromal cell-seeded scaffolds. **d** Growth-competent stromal cells compactly filled with fibroblastic cells. **e** Growth-arrested stromal cells formed organized tissue microenvironments that accommodate both stromal and hematopoietic components. Both growth competent and growth arrested hTNFa stromal cell-scaffolds showed minimum stromal component and mimicked inflammatory microenvironment. (Scale bar, 200 μm)

The next aspect of our study was to understand local tissue development in scaffolds that were seeded with growth-arrested stromal cells compared to growth-competent stromal cells. Gross images of explanted scaffolds seeded with growth-competent engineered stromal cells showed excessive tissue development that completely engulfed the implanted scaffolds except the hTNFa scaffolds (Fig. 3a, b). On the other hand, growth-arrested stromal cell scaffolds maintained their original dimension and showed dark red color due to locally recruited red blood cells (Fig. 3c). Histopathological analysis of growth-competent stromal cell scaffolds revealed a densely populated stromal cells and deposited extracellular matrix (ECM) with few hematopoietic cells, while hTNFa scaffolds were mostly filled with hematopoietic cells that mimics tissue inflammation (Fig. 3d). In case of growth-arrested stromal cell scaffolds, developed tissues were hypocellular and accommodating both stromal and hematopoietic components in an organized manner. Hematopoietic cells were primarily found at the pore surface and stromal cells were located in the pore center. Control and hVEGFa scaffolds showed comparable microenvironments, while hSDF1a scaffolds exhibited significantly increased hematopoietic cells. Again hTNFa scaffolds showed partially acellular areas with an inflammatory leukocyte reaction (Fig. 3e). Further SEM analysis distinguished densely filled fibroblastic tissue and ECM in growth-competent stromal cell-scaffolds, and co-existence of hematopoietic and stromal cells in growth-arrested stromal cell-scaffolds (Additional file 1: Figure S4).

### Implanted microenvironments support systemic migration and retention of human CD34 cells

**Fig. 4:**
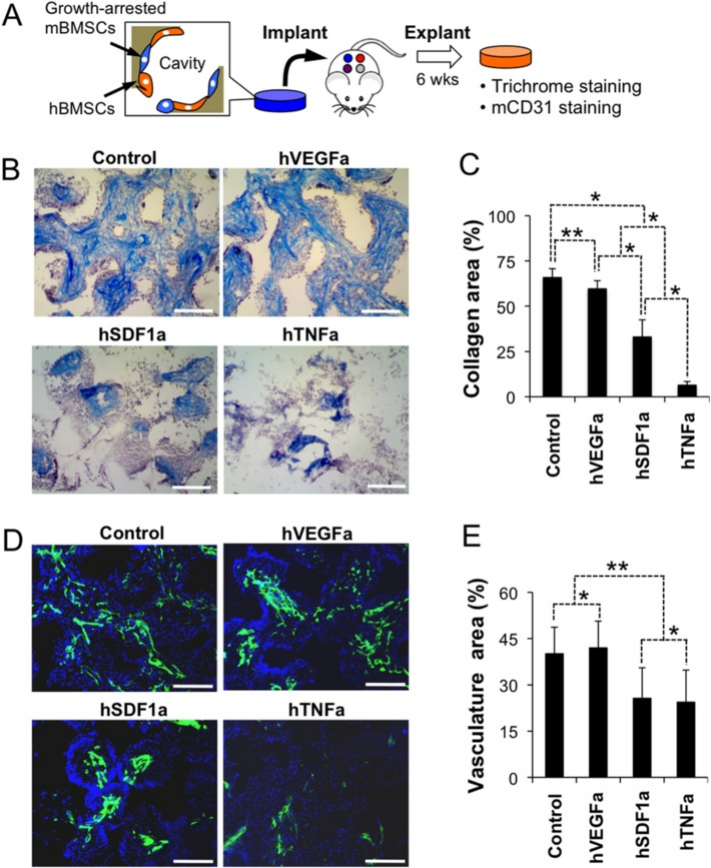
Analysis of growth-arrested engineered stromal cell type dependent tissue microenvironments. **a** Schematic of experiment. Growth-arrested genetically engineered stromal cells were co-seeded with hBMSCs into hydrogel scaffolds and subdermally implanted for 6 weeks. Explanted scaffolds were characterized for collagen deposition and vasculature development. **b** Masson’s Trichrome staining visualizes collagen fibers (Blue). **c** Semi-quantitative analysis of collagen deposited area (*N* = 6). **d** Immunohistostaining of mouse CD31 (green) and nucleus (DAPI) identifies inter-scaffold vasculatures. **e** Semi-quantitative analysis of vasculature area (*N* = 6). (Scale bar, 200 μm) (*P < 0.01, **P < 0.05)

Having multiple, autonomous microenvironments in a single mouse provided an opportunity to study human HSPCs function in unique ways. In our next set of experiments, we exploited growth-arrested stromal cell scaffolds that form locally enriched human soluble microenvironments while providing room for growth of human HSPCs. Prior to seeding growth-arrested stromal cells were mixed with hBMSCs that improved survival of stromal cells after in vivo implantation in the above study (Fig. 4a). We first characterized soluble factor-dependent tissue microenvironments focusing on inter-scaffold ECM deposition and angiogenesis. Trichrome staining and semi-quantitative image analysis revealed significantly enhanced collagen deposition in control and hVEGFa scaffolds, whereas only sparse collagen fibers were deposited in hTNFa scaffolds reminiscence to a necrotic tissue (Fig. 4b, c and Additional file 1: Figures S5 and S6). Immunohistostaining of mouse CD31 (mCD31) endothelial cells showed considerably more blood vessels in control and hVEGFa scaffolds than hSDF1a and hTNFa scaffolds (Fig. 4d and e). Collectively these results indicate that growth arrested stromal cells created a soluble factor enriched local microenvironments that induced distinct tissue formation.

**Fig. 5:**
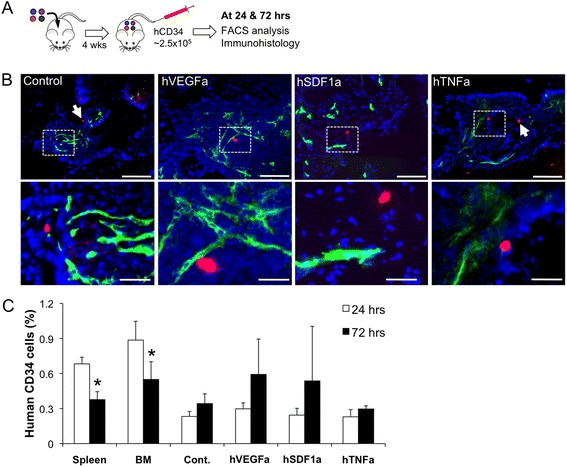
Systemic recruitment of intravenously delivered hCD34 cells to the implanted tissue microenvironments. **a** Schematic of experimental design for systemic migration of hCD34 cells to the different types of genetically engineered stromal cell-laden scaffolds, **b** Immunohistostaining of hCD34 and mCD31 cells of frozen tissue sections prepared post 72 h hCD34 cell injection. Arrows indicate hCD34 cells. (Red: hCD34, Green: mCD31 and Blue: nucleus/Scale bars, 200 μm for upper and 50 μm for lower images) (**c**) Flow cytometric analysis of hCD34 cells post 24 and 72 h intravenous injection. (*N* = 3, *P < 0.05)

We next evaluated if human CD34 (hCD34) HSPCs migrate to specific engineered implants in a competitive transplantation assay. In a single mouse, 4 different types stromal cell seeded scaffolds were implanted and matured in vivo for 4 weeks. Mice were administered 2 × 10^5^ hCD34 cells by intravenous injection. At 24 and 72 h post-injection, we explanted scaffolds and retrieved hematopoietic cells to measure short-term migration of hCD34 cells to the scaffolds (Fig. 5a). Immunohistostaining of explanted scaffolds post 72 h injection confirmed that implanted microenvironments recruited circulating hCD34 cells. Most hCD34 cells were detected next to the vasculature as single cells and their number was comparable among stromal cell types (Fig. 5b). Flow cytometric analysis quantified the percentage of migrated hCD34 cells to the implanted scaffolds, which was significantly lower when compared to the endogenous bone marrow and spleen. The percentage of hCD34 cells remained the same in control and hTNFa scaffolds and even increased in hVEGFa and hSDF1a scaffolds between 24 and 72 h characterization. On the other hand, hCD34 cell number in the bone marrow and spleen were reduced after 72 h (Fig. 5c). In general, an implanted microenvironment recruited circulating hCD34 cells and retained them, though differential migration to a preferential scaffold was not observed in this model system.

### Skewed in vivo differentiation of hCD34 cells in engineered microenvironments

**Fig. 6:**
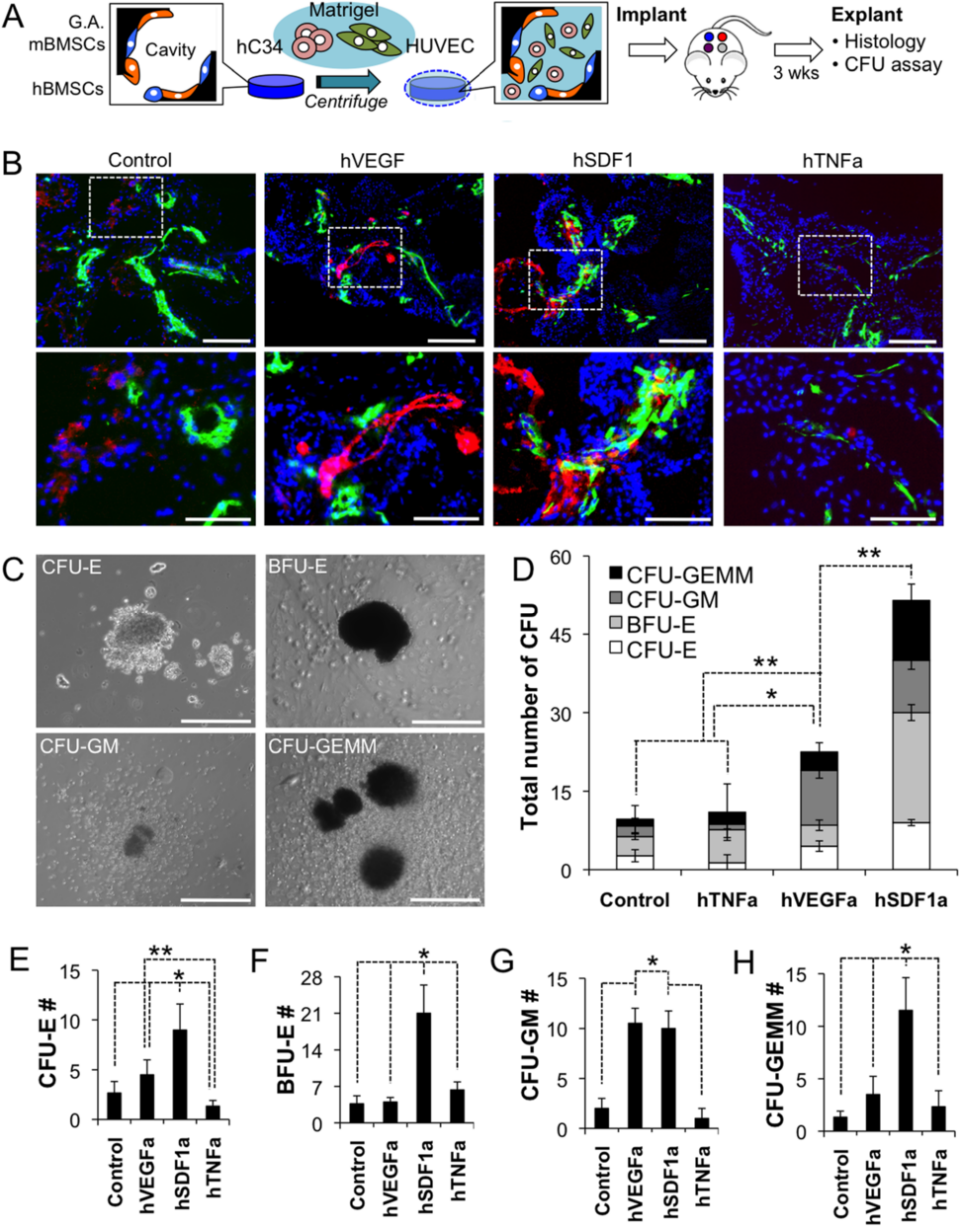
Analysis of soluble microenvironments dependent hCD34 cell behavior after co-implantation with hBMSCs and genetically engineered mBMSCs. **a** Schematic of experimental procedure. Once hBMSCs and growth-arrested genetically engineered mBMSCs formed stable adhesion on pore surface, the pore space was filled with hCD34 and HUVECs dispersed in liquid state ice-cold matrigel. Matrigel was subsequently solidified and hCD34 cell-retaining scaffolds were implanted to NSG mice for 3 weeks. **b** Immunohistostaining of explanted scaffolds post 3 weeks implantation. (Green: mCD31, red: hCD34, and blue: nucleus/Scale bar, 200 μm). **c** Representative images of colony forming units after 14 days methylcellulose plating (Scale bar, 300 μm), **d** Compression of total CFU counting in different stromal-cell implants. **e**-**f** Comparison of specific CFU counting for differentiated hematopoietic cell types; (**e**) CFU-Erythroid (CFU-E), (**f**) Burst Forming Unit-Erythroid (BFU-E), (**g**) CFU-Granulocyte and Macrophage (CFU-GM), (**h**) CFU-Granulocyte, Erythrocyte, Monocyte, and Megakaryocyte (CFU-GEMM). (*N* = 3, *P < 0.5, **P < 0.05)

The soluble factors that were selected for overexpression in stromal cells all have known effects on HSPCs migration, differentiation, and maintenance [22–24]. In this subset study, we wanted to verify the bioactivity of these molecules on differentiation in our model system. Scaffolds pre-seeded with growth-arrested genetically engineered stromal cells and hBMSCs were cultured for 3 days. On the day implantation, hCD34 cells and HUVECs dispersed in matrigel were infiltrated into the cavities of hydrogel scaffolds by centrifugation and then solidified resulting in a complex 3D tissue structure that was durable throughout the implantation procedure (Fig. 6a). HUVECs were used to induce rapid angiogenesis in the implanted microenvironments for promoting the survival of hCD34 cells since they were directly implanted into a non-vascularized scaffold in this study. This tissue construct was implanted into mice at 4 different locations representing the one different engineered soluble factor that was overexpressed. After 3 weeks of in vivo growth, the scaffolds were explanted and stained for hCD34 and mCD31 expressing cells. The results reveled that hCD34 cells were still present in the scaffold at this time point and located in close proximity with vasculatures in all scaffolds, except hTNFa scaffolds (Fig. 6b). Separately, scaffold cells were retrieved and put in a colony forming unit (CFU) assay to assess the number of progenitor cells and their skewed differentiation potential. Isolated cells from each environment showed visual signs of erthyroid, granulocyte, monocyte, macrophage, and megakaryocyte by 14 days of differentiation culture in methylcellulose with supplemented human growth factors and cytokines. Isolated cells from each environment showed visual signs of erthyroid, granulocyte, monocyte, macrophage, and megakaryocyte by 14 days of differentiation culture in methylcellulose with supplemented human growth factors and cytokines (Fig. 6c). Total CFU counting result distinguished that hSDF1a scaffolds retain substantially higher number of hematopoietic progenitor cells followed by hVEGFa scaffolds. Control and hTNFa scaffolds accommodate comparable level, low number of hematopoietic progenitor cells (Fig. 6d). Further enumeration of specific colonies that were grown from each stromal cell type, revealed a very potent effect of hSDF1a on the maintenance of a progenitor pool in all lineages (Fig. 6e-h). Other major notable findings were the reduction of erythroid progenitors by hTNFa (Fig. 6e) and the positive effects of hVEGFa overexpression on the number of granulocyte/macrophage progenitor populations (Fig. 6g). The results show that this overexpression system produces bioactive human molecules that have differentiation effects on the human hematopoietic system as corroborated by several previous studies [25–28].

## Discussion

The goal of humanized mouse models is to reconstitute specific cellular and tissue dynamics to study human cell biology in vivo and potentially become a testbed to predict human responses for precision medicine. Studies using immunodeficient mice that accept human cells/tissue have generally shown that by adding complimentary human tissues, a more humanized response can be seen [10]. In this study, we wanted to create a testing platform where molecules of interest to the human hematopoietic system can be studied in a locally enriched environment with easy access and analytical capability. Our hypothesis was that a single mouse can harbor multiple independent microenvironments that have site-specific bioactivity by implanting genetically engineered stromal cell-laden scaffolds. Mouse cells are generally more difficult to infect with lentivirus than human cells, though they are more proliferative after infection and also could survive longer after in vivo implantation than human cells [29]. Our infection efficiency of mouse stromal cells was ~10–30 %. The transfected cells maintained proliferative capacity after purification by flow sorting and expressed properly folded hVEGFa, hSDF1a, and hTNFa. The constitutive translation of these proteins by engineered cells was 4–5 fold more in concentration than normal hBMSCs with respect to hVEGFa and hSDF1a. hTNFa is not naturally expressed by hBMSCs and this construct was included to mirror an inflammatory reaction site. This mouse cell expression was robust and took only a few weeks as opposed to months/years for transgenic mice, which can further benefit from improvement in infection efficiency while exploring new promoter systems and unknown molecules of interest to microenvironment research.

When compared to in vivo gene delivery methods that non-specifically infect host cells to synthesize desired human cytokines, an engineered ectopic tissue analogue is an alternate approach with multiple advantages. First, the porous hydrogel scaffolds were a very useful tool for standardizing and analyzing engineered tissue microenvironments. The fully interconnected microscale cavities made with a synthetic hydrogel matrix promoted angiogenesis and, in turn, improved the survival and function of genetically engineered stromal cells after implantation. These features allowed for direct comparison of the effects of the overexpressed gene on local tissue development and human HSPC fate. Second, these engineered tissue analogues could be studied for local or systemic effects by simply controlling the growth potential of the engineered stromal cells. This also enables an increase in the throughput of studies by implanting several unique microenvironments into a single host mouse. Finally, implanted microenvironments can accommodate human HSPCs and be directly accessed for various imaging and analytical tools [20, 30]. Thus, implantable microenvironments that harbor genetically engineered cells can broaden the study of human cells in immunodeficient mouse models.

In our in vivo studies with engineered stromal cells, we observed systemic levels of expressed cytokine and a mild sarcoma-like overgrowth of these stromal cell lines that were not under growth arrest. A typical fibroblastic compartment is quiescent, unless the tissue bed is activated at which time the cells have been observed to proliferate. The tissue cavity was not considered conducive to support primary functions of hematopoiesis. Instead, a growth-arrested stromal cell population could still express soluble, bioactive factors in an enriched environment while allowing for additional tissue elements (e.g. hBMSCs, HUVECs, hCD34 cells) to be included and maintain viability within the space as seen in Fig. 5. Using growth-arrested stromal cells eliminated systemic levels of expressed cytokines as well. This stromal population was put on test in a competitive transplantation assay and we did not observe site-specific homing of hCD34 cells, even to a potent chemokine hSDF1a. The lack of a systemic chemokine gradient may be a plausible explanation for non-specific homing results and can be an area of improvement with a temporal and stronger burst of cytokine from a non-proliferative cell mass.

The human cytokines overexpressed in our model system, namely hVEGFa, hSDF1a, and hTNFa, were selected based on known biological responses to hematopoietic activity to benchmark our bioactivity studies. hVEGFa is a potent stimulator of angiogenesis and has direct effect on promoting the survival of HSPCs [31, 32]. hSDF1a is a well-known chemokine molecule that actively recruits circulating HSPCs [27, 33, 34] and maintains their activities [35]. hTNFa restricts HSPCs activity functioning as a key precursor for inflammatory response [36, 37]. Although these cytokines are designed for human specific, evolutionary conservation of key structures and functions in these molecules exhibited cross-reactivity with murine tissue/cells that in turn promote distinct tissue microenvironment formation depending on types of engineered stromal cells. We observed sustained vascular area in hVEGFa constructs, more cellularity and progenitor cell activity in hSDF1a constructs, and more inflammatory cell recruitment and collagen remodeling in hTNFa constructs. Our model system further reflected key biological responses in vivo interacting with hCD34 HSPCs. This model system can be deemed suitable to understand short-term effects of engineered human proteins on HSPCs function for future discovery and validation studies. There may also be cross-reactivity of mouse molecules on human HSPCs and vice versa, though we maintained the same cellular composition of our scaffolds to control for this potential effect. A long-term evaluation of these microenvironments is necessary to establish true stem cell functions of serial transplantation, reconstitution, and tri-lineage differentiation after months in vivo.

## Conclusion

In conclusion, we presented a bioengineering strategy to improve humanized mouse models by creating implantable microenvironments that release human specific cytokines and growth factors in a controlled manner. Genetically engineered stromal cells stably synthesize encoded human molecules after in vivo implantation. Porous hydrogel scaffolds facilitated the usage of engineered stromal cells as forming tissue-engineered depots that continuously release engineered human molecules. Presented approach is simple and versatile that can be easily applied to other soluble and insoluble molecules. Combination of engineered stromal cells and implantable microenvironments is expected to be a valuable tool for generating humanized mouse models and utilizing them for basic and translational human HSPCs as well as cancer research.

## Abbreviations

hVEGFa, vascular endothelial growth factor a; hSDF1a, stromal derived factor 1 alpha; hTNFa, tumor necrosis factor alpha; HSPCs, hematopoietic stem and progenitor cells; hBMSCs, human bone marrow stromal cells; mBMSCs, mouse bone marrow stromal cells; HUVECs, human umbilical vein endothelial cells; a-MEM, alpha-minimum essential medium; FACS, fluorescence activated cell sorting; SEM, scanning electron microscopy; ECM, extracellular matrix; CFU, colony forming unit; NSG, NOD-scid IL2rγ^null^; eGFP, enhanced green fluorescent protein; MHC, major histocompatibility complex; MTT, MTT 3-(4,5-dimethylthiazol-2-yl)-2,5-diphenyltetrazolium bromide

## Additional file

Additional file 1: Figure S1. Selection and conformation of lentivial transfected mouse stromal cells. (A) Flow cytometric analysis of GFP mBMSC, (B) Culture-expanded genetically engineered mBMSCs. (Scale bar, 200μm). **Figure S2.** Characterized secretion of human cytokines from genetically engineered stromal cells in 1 and 3 weeks in vitro culture. **Figure S3.** hSDF1a ELISA in mouse blood serum. Control mice without scaffold implantation showed a background level of SDF1a signal due to cross-reactivity. This level was used as a baseline and was also observed in growth arrested, which was concluded, as undetectable. The other groups showed measurable levels above background and were concluded to be true hSDF-1a detection. **Figure S4.** SEM images of growth-competent genetically engineered stromal cell-seeded scaffolds. (A) Cross-sectional images of human soluble factor secreting engineered stromal cell-seeded scaffolds after 6 weeks subcutaneous implantation. Except hTNFa, entire pores were completely filled with tissue cells with no hematopoietic components. (B) Closed-up image of growing engineered stromal cell-seeded scaffolds. **Figure S5.** Examples of semi-quantitative image analysis using ImageJ. (A) Collagen fiber area estimation from a Masson’s Trichrome staining image, (B) Vasculature area estimation from an immunohistostaining mCD31 and DAPI image. **Figure S6.** Long-term maintenance of inflammation-mimicking tissue microenvironment indirectly indicates survival and function of growth-arrested hTNFa secreting engineered stromal cells in the implanted scaffolds. (DOCX 2962 kb)
