# Supplementary material for: Multiple genetically engineered humanized microenvironments in a single mouse
Source: Biomater Res. 2016 Jun 28;20:19. doi: 10.1186/s40824-016-0066-2 (PMC4924259; doi:10.1186/s40824-016-0066-2)
Supplement: Additional file 1: Figure S1. — Selection and conformation of lentivial transfected mouse stromal cells. (A) Flow cytometric analysis of GFP mBMSC, (B) Culture-expanded genetically engineered mBMSCs. (Scale bar, 200μm). Figure S2. Characterized secretion of human cytokines from genetically engineered stromal cells in 1 and 3 weeks in vitro culture. Figure S3. hSDF1a ELISA in mouse blood serum. Control mice without scaffold implantation showed a background level of SDF1a signal due to cross-reactivity. This level was used as a baseline and was also observed in growth arrested, which was concluded, as undetectable. The other groups showed measurable levels above background and were concluded to be true hSDF-1a detection. Figure S4. SEM images of growth-competent genetically engineered stromal cell-seeded scaffolds. (A) Cross-sectional images of human soluble factor secreting engineered stromal cell-seeded scaffolds after 6 weeks subcutaneous implantation. Except hTNFa, entire pores were completely filled with tissue cells with no hematopoietic components. (B) Closed-up image of growing engineered stromal cell-seeded scaffolds. Figure S5. Examples of semi-quantitative image analysis using ImageJ. (A) Collagen fiber area estimation from a Masson’s Trichrome staining image, (B) Vasculature area estimation from an immunohistostaining mCD31 and DAPI image. Figure S6. Long-term maintenance of inflammation-mimicking tissue microenvironment indirectly indicates survival and function of growth-arrested hTNFa secreting engineered stromal cells in the implanted scaffolds. (DOCX 2962 kb) [file 40824_2016_66_MOESM1_ESM.docx]

Additional file 1

**Multiple genetically engineered humanized microenvironments in a single mouse**

Jungwoo Lee^1,4*^, Dirk Heckl^2^, Biju Parekkadan^1,3*^

1. Department of Surgery, Center for Engineering in Medicine, Massachusetts General Hospital & Harvard Medical School and Shriners Hospital for Children, USA

2. Department of Medicine, Brigham and Women’s Hospital, USA

3. Harvard Stem Cell Institute, USA

4. Department of Chemical Engineering, Institute for Applied Life Sciences, University of Massachusetts, USA

Correspondence: [jungwoo@engin.umass.edu](mailto:jungwoo@engin.umass.edu), [biju_parekkadan@hms.harvard.edu](mailto:biju_parekkadan@hms.harvard.edu).

| **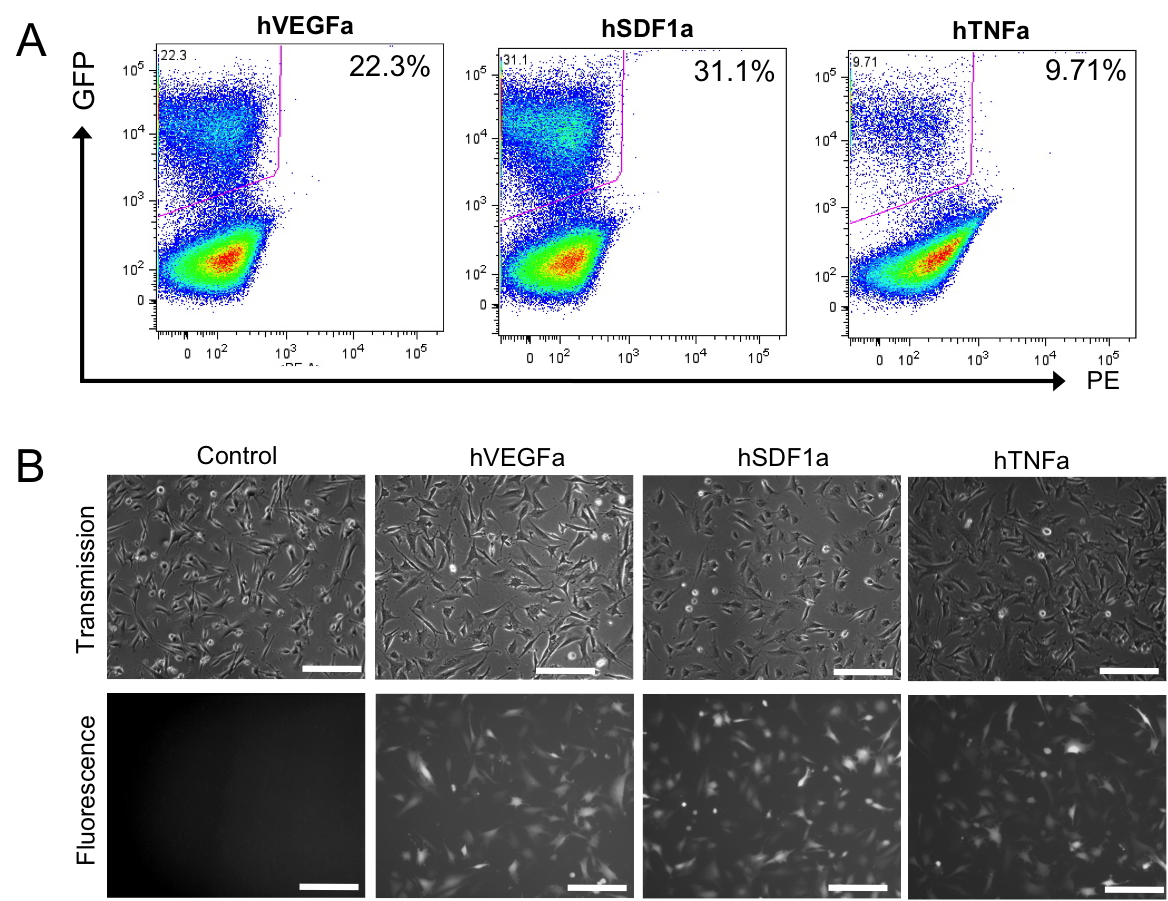** |
| --- |
| **Figure S1. Selection and conformation of lentivial transfected mouse stromal cells. (A)** Flow cytometric analysis of GFP mBMSC, **(B)** Culture-expanded genetically engineered mBMSCs. (Scale bar, 200µm) |

| **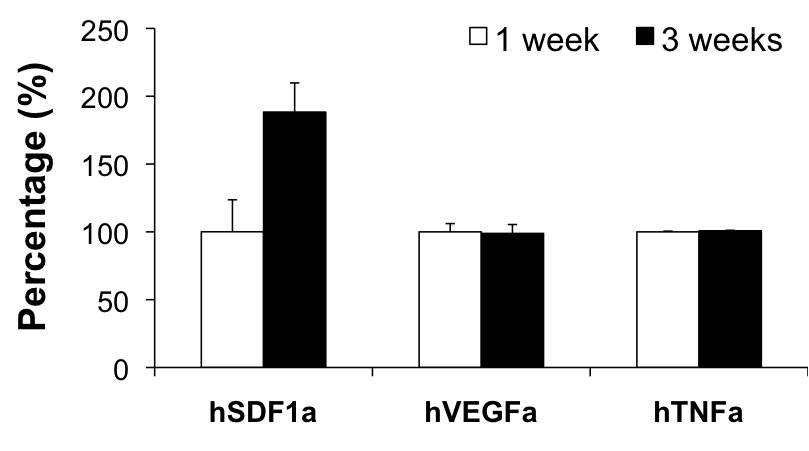** |
| --- |
| **Figure S2.** Characterized secretion of human cytokines from genetically engineered stromal cells in 1 and 3 weeks *in vitro* culture. |

| **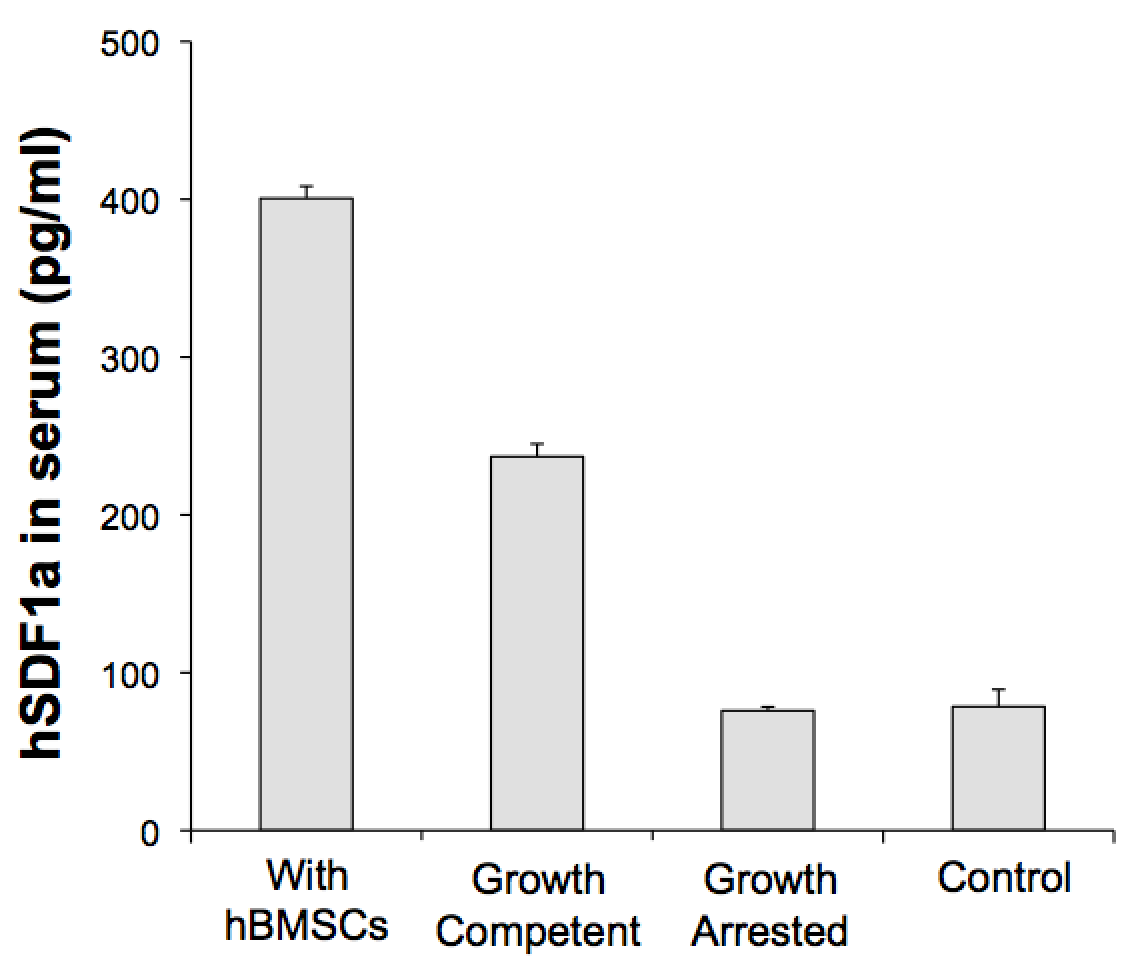** |
| --- |
| **Figure S3.** hSDF1a ELISA in mouse blood serum. Control mice without scaffold implantation showed a background level of SDF1a signal due to cross-reactivity. This level was used as a baseline and was also observed in growth arrested, which was concluded, as undetectable. The other groups showed measurable levels above background and were concluded to be true hSDF-1a detection. |

| 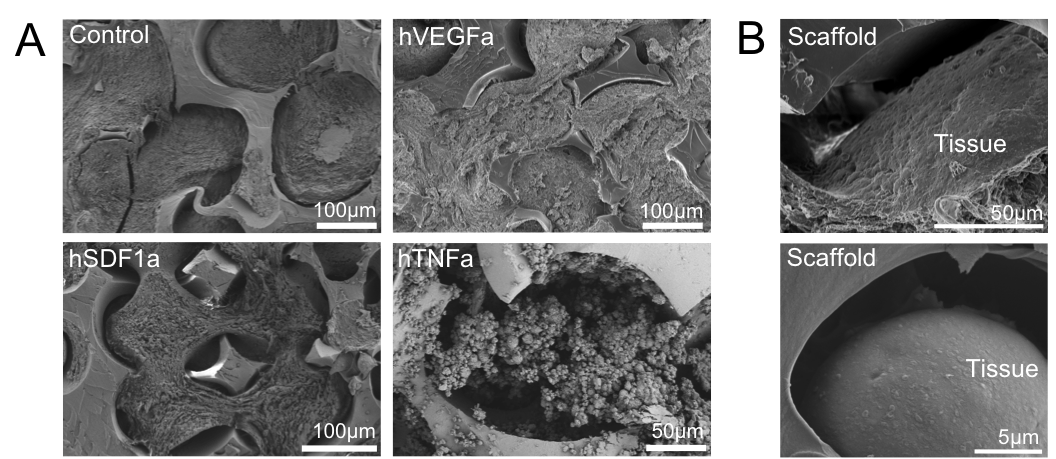 |
| --- |
| **Figure S4. SEM images of growth-competent genetically engineered stromal cell-seeded scaffolds. (A)** Cross-sectional images of human soluble factor secreting engineered stromal cell-seeded scaffolds after 6 weeks subcutaneous implantation. Except hTNFa, entire pores were completely filled with tissue cells with no hematopoietic components. **(B)** Closed-up image of growing engineered stromal cell-seeded scaffolds. |

| **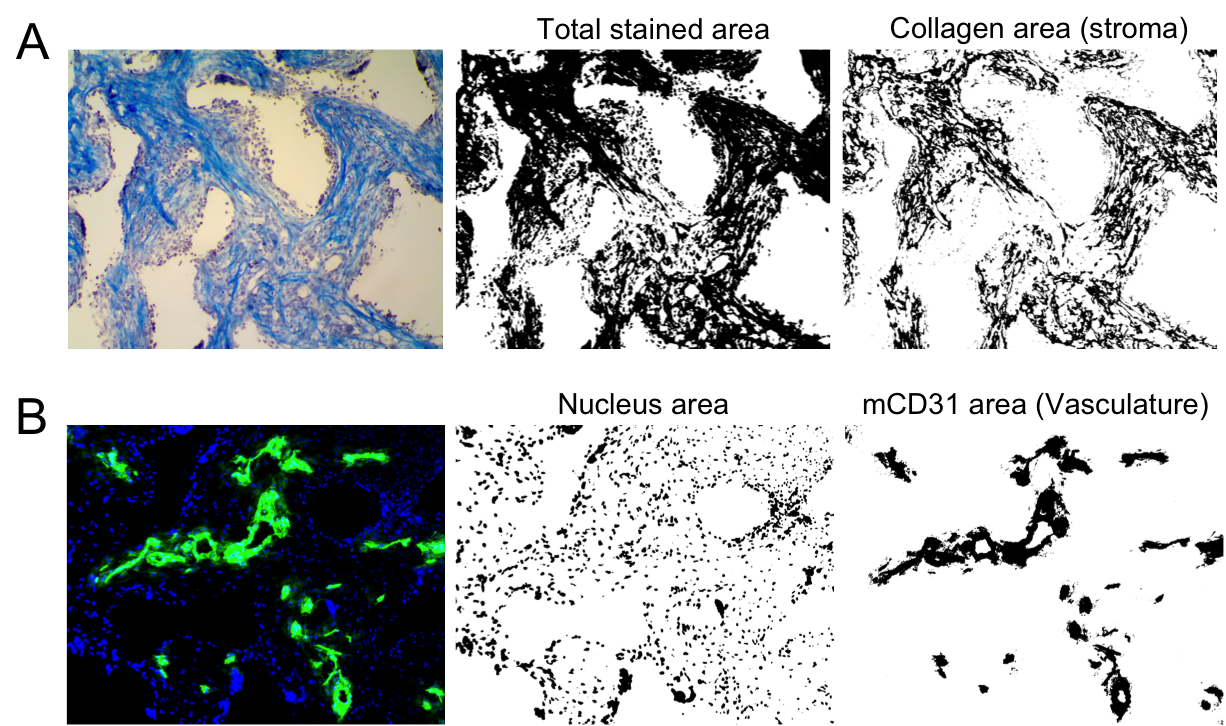** |
| --- |
| **Figure S5. Examples of semi-quantitative image analysis using ImageJ. (A)** Collagen fiber area estimation from a Masson’s Trichrome staining image, **(B)** Vasculature area estimation from an immunohistostaining mCD31 and DAPI image. |

| **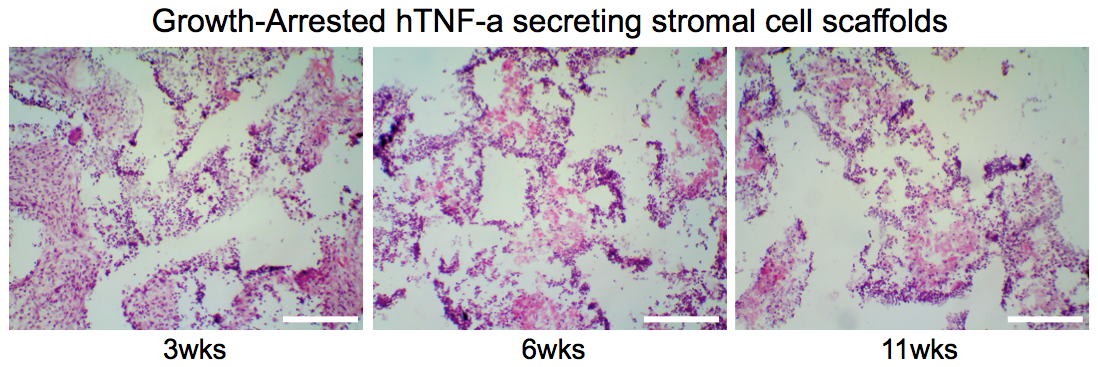** |
| --- |
| **Figure S6.** Long-term maintenance of inflammation-mimicking tissue microenvironment indirectly indicates survival and function of growth-arrested hTNFa secreting engineered stromal cells in the implanted scaffolds. |
